# HPV Status and second primary tumours in Oropharyngeal Squamous Cell Carcinoma

**DOI:** 10.1186/1916-0216-42-36

**Published:** 2013-05-29

**Authors:** Caroline C Xu, Vincent L Biron, Lakshmi Puttagunta, Hadi Seikaly

**Affiliations:** 1 Division of Otolaryngology-Head and Neck Surgery, Department of Surgery, University of Alberta Hospital, 8440 112 St, Edmonton, AB T6H 2H7, Canada; 2 Department of Laboratory Medicine and Pathology, University of Alberta Hospital, 8440 112 St, Edmonton, AB, T6H 2H7, Canada

**Keywords:** Oropharynx, Second primary tumours, Panendoscopy, HPV, P16

## Abstract

**Introductions:**

The incidence of human papillomavirus (HPV)-related oropharyngeal squamous cell carcinoma (OPSCCs) is rising in developed nations. Studies have shown that these virally mediated tumours are epidemiologically, clinically, and biologically different than other head and neck squamous cell carcinomas and traditional concepts of field cancerization may not apply to HPV-related oropharyngeal cancer.

**Objective:**

The purpose of this study was to evaluate the rate of second primary tumors and the diagnostic yield of field cancerization work up in the upper aerodigestive tract in patients with HPV-related and HPV-unrelated oropharyngeal squamous cell carcinoma.

**Design:**

Retrospective review.

**Setting:**

Tertiary cancer care centers in Alberta.

**Methods:**

Retrospective review of 406 patients diagnosed with OPSCC in Alberta between 2005 and 2009. HPV-status of tumours was determined by tissue microarray using immunohistochemistry staining for p16.

**Main outcome measures:**

Primary outcome: incidence of upper aerodigestive tract second primary tumours in p16-positive versus p16-negative OPSCC. Secondary outcomes: diagnostic yield of traditional field cancerization work-up in p16-positive versus negative patients.

**Results:**

The overall rate of SPTs was 7.4% (30/406). The incidence rate of SPTs was significantly lower in p16-positive patients (0.7 per 100 patient-yrs vs. 8.5 in p16-negative, p < 0.0001). Field cancerization work-up for synchronous lesions in the upper aerodigestive tract, including panendoscopy and whole-body PET-CT, had decreased diagnostic yield in p16-positive patients (2.8% vs. 10.2% in HPV-negative patients, p=0.02).

**Conclusions:**

Patients with HPV-related OPSCC, who are non-smokers have decreased risk of developing second primary tumours in the upper aerodigestive tract and have low yield on field cancerization work-up. This study provides further evidence that virally mediated OPSCC are distinct and may benefit from alternate diagnostic pathways.

## Introduction

The incidence of human papillomavirus (HPV)-related oropharyngeal squamous cell carcinoma (OPSCCs) is rising in developed nations [1-4]. Studies have shown that these virally mediated tumours are epidemiologically, clinically, and biologically different than other head and neck squamous cell carcinomas [5-7]. For example, a recent study from our institution demonstrated a strong epidemiologic relationship between OPSCC in women and human papillomavirus-associated cancers [8]. These differences are highlighted by the fact that HPV-positive OPSCC patients have improved clinical outcomes and overall survival after tratment [9,10].

The concept of ‘field cancerization’ has been described in head and neck squamous cell carcinoma (HNSCC) with traditional risk factors such as alcohol and tobacco. It proposes that HNSCCs have a high propensity for local recurrences and second primary tumours due to a large pre-neoplastic field of mucosal epithelium exposed to carcinogens. Molecular studies have shown that this epithelium contains cells that have alterations of the PI3K-PTEN-AKT pathway [10]. This pathway ultimately results in perturbed p53 and retinoblastoma (RB) pathways, both of which are broadly implicated in carcinogenesis [11]. The concept of field cancerization is the basis for most of our diagnostic and follow up protocols for HNSCC patients. This includes routine panendoscopy and PET-CT scanning in many centers for detection of second primary tumours in this musocal field [12,13].

HPV-mediated carcinogenesis also occurs through inactivation of p53 and retinoblastoma, but through expression of two viral oncogenes: E6 and E7 [11,14]. This also results in the overexpression of p16, which has been widely accepted as a surrogate marker for HPV infection. Critical events in this process include viral infection of a single cell followed by monoclonal expansion and replication. Given the molecular differences in HPV-mediated carcinogenesis, it is reasonable to hypothesize that traditional concepts of field cancerization may not apply to HPV-related oropharyngeal cancer. This concept is confirmed by recent epidemiologic data showing lower rate of second primary malignancy after an index OPSCC, despite an increasing overall incidence of HPV-induced HNSCC worldwide, particularly in healthy, young males [4].

The purpose of this study is two fold:

1) To evaluate the rate of second primary tumours in the upper aerodigestive tract in patients with HPV-related and HPV-unrelated oropharyngeal squamous cell carcinoma.

2) To assess the diagnostic yield of field cancerization work up for second primary tumours in the upper aerodigestive tract in patients with HPV-related and HPV-unrelated oropharyngeal squamous cell carcinoma.

## Methods

The University of Alberta Human Research Ethics Board approval was obtained for this study. A retrospective review was conducted of 406 consecutive patients treated for OPSCC in Alberta between 2004 and 2009, inclusive. Patients were identified through the Alberta Cancer Board database of head and neck cancer patients. Patient and tumour demographic data, follow-up and survival data were recorded. Smoking status was also recorded for all patients. These were based on self-reported tobacco use at the time of initial assessment at the Cross Cancer Institute (CCI, Edmonton, AB) or Tom Baker Cancer Centre (TBCC, Calgary, AB). Patients were considered non-smokers only if life-long non-smoking status was reported.

### Identification of second primary tumours

Second primary tumours were identified by review of CCI or TBCC clinical progress notes, whole-body PET-CT imaging reports, and histopathologic reports. Lesions were considered second primary tumours if they were at least > 2cm distal from the index malignancy and histologically proven to be inconsistent with recurrence or metastatic disease. All second primary tumours were classified as synchronous if identified within 6 months of diagnosis the primary tumor and metachronous if identified outside of this 6-month period. Upper aerodigestive tract (UADT) sites included all head and neck subsites, esophageal, and lung cancers. All other tumours were considered non-upper aerodigestive tract (Non-UADT) and included colorectal, breast, prostate, and thyroid malignancies.

### Incidence rates

The incidence rate (IRs; events per 100 patient-yrs) and their confidence intervals were calculated. Observation time was defined as time from initial diagnosis to last date of follow-up or death.

### Determination of HPV status

Of the 406 patients, 199 formalin-fixed and paraffin-embedded tissues were able to be retrieved from the Alberta tumour bank and prepared for tissue microarray as previously described [15]. Each sample was incubated with a p16^INK4a^ mouse monoclonal antibody (p16) and visualized using avidin-biotin-peroxidase technique. Positive p16 status was defined as high-intensity, diffuse staining of more than 70% per spot. This was digitally scored using standardized cutoffs with AQUAnalysis software (HistoRx, Inc. Branford, Conneticut).

### Yield on field cancerization work-up for synchronous SPTs

All the investigations for second primaries usually performed in the cancer centers were reviewed. Diagnostic yield was defined as the number of SPTs identified by a diagnostic test divided by the number of individuals undergoing the diagnostic test.

### Statistical and multivariate analysis

All analysis was completed using SPSS 19 (Chicago, IL). Comparison of parametric data was by unpaired t-test and non-parametric, categorical data were compared by Fisher’s exact test. Two by two contingency tables were generated for comparisons of categorical data and Fisher’s exact test was used to test significance.

## Results

### Patient characteristics

406 patients were treated for OPSCC between 2005 and 2009. P16 staining was completed for 49.1% of patients. The demographics and distribution of patients by p16 status is shown in Table 1. Patients with known p16 status were younger, more male-predominant and fewer were smokers.

**Table 1:** Summary of patient demographic information by p16 status

		**All patients (N= 406)**		**p16 status available (N = 199)**		
				**p16 + (N=118)**	**p16 - (N=81)**	**p-value**
Average age		59.1	±10.9	55.9	60.7	0.003*
Sex	Male	327	79.8%	86.6%	76.3%	0.047*
	Female	83	20.2%			
Smokers		281	68.5%	63.5%	86.8%	0.0008*
Stage	IV	308	78.8%	75.6%	80.0%	0.49
	III	58	14.2%	18.5%	11.3%	0.23
	II	26	6.3%	2.5%	3.7%	0.68
	I	14	3.4%	1.7%	2.5%	0.99
Index tumour location	Tonsils	213	68.7%			
	Base of the tongue	90	29.0%			
	Oropharynx NOS	15	4.8%			
	Soft palate	18	5.8%			
	Pharyngeal walls	15	4.8%			
Treatment	Surgery+ChemoRT	143	34.9%			
	ChemoRT	216	52.7%			
	Palliative	16	3.9%			
	Salvage surgery	11	2.7%			
	No treatment	21	5.1%			
Follow-up (years)		3.9	± 1.0	3.9	3.9	0.98

p < 0.05 considered statistically significant. NOS = not otherwise specified.

### Upper aerodigestive tract second primary tumours

The overall prevalence of upper aerodigestive tract (i.e. head and neck, esophageal, and bronchogenic) SPTs was 7.39%. The SPT incidence rates per 100 patient-years were significantly reduced in p16-positive OPSCC patients (Table 2). In contrast, rates of non-upper aerodigestive tract second primaries were not significantly different between p16 positive and negative patients (Table 2).

**Table 2:** Incidence rate of upper aerodigestive tract (UADT) and non-upper aerodigestive tract (non-UADT) second primary tumours by p16 status

	**Incidence rate per 100 patient-yrs (Confidence interval)**		
	**p16 +**	**p16 -**	**p-value**
UADT	0.7	8.5	<0.0001*
	(0.3 – 1.4)	(5.7 – 12.4)	
Non-UADT	0.9	2.1	0.116
	(0.9 – 4.7)	(0.4 – 1.8)	

p < 0.05 considered statistically significant.

**Figure 1:**
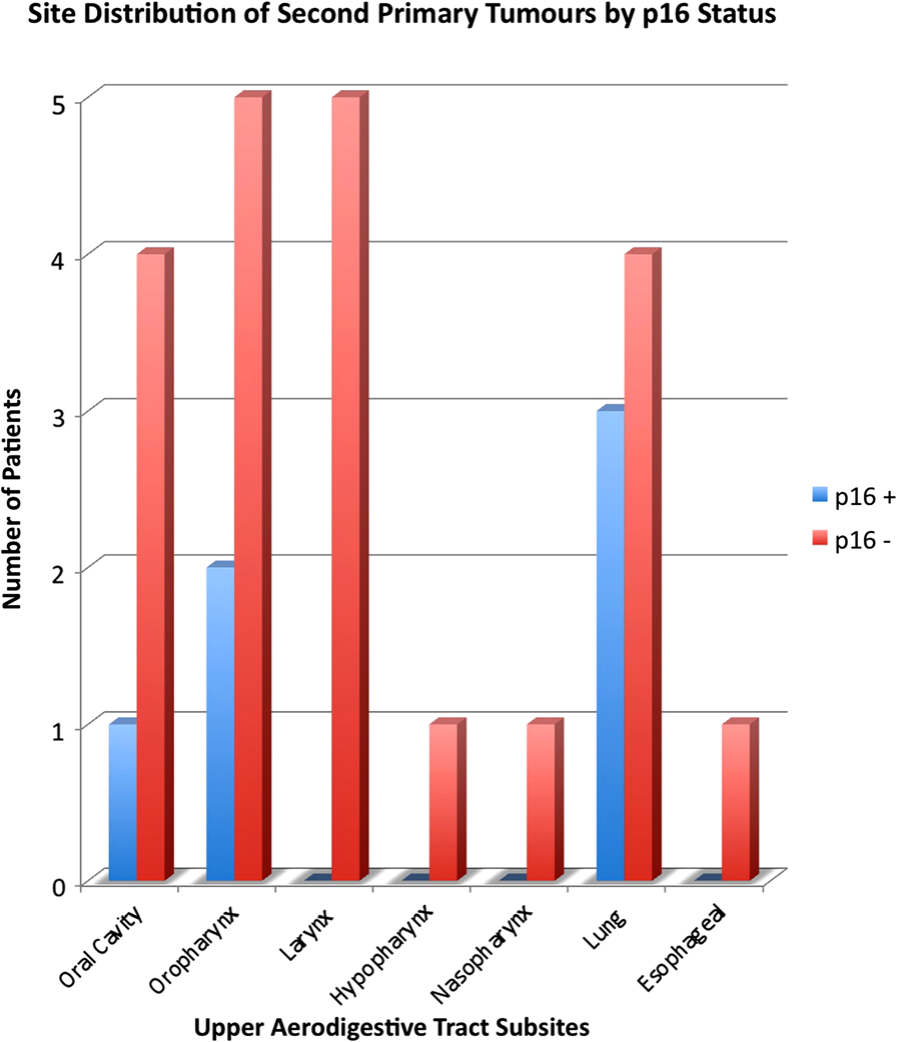
Site distribution of second primary tumours by p16 status.

The distribution of upper aerodigestive tract SPTs differed between p16-positive and p16-negative OPSCC (Figure 1). Patients with p16-positive OPSCC had SPTs in the oral cavity, tonsil, and lung. Specifically, lung lesions comprised of adenocarcinomas. Non-HPV-related OPSCC patients had SPTs distributed across all subsites of UADT.

### Yield on field cancerization work-up for synchronous SPTs

Sixty-four p16-positive patients underwent whole-body PET-CT scanning and 80 underwent panendoscopy within six months of the initial diagnosis of their index head and neck malignancy. Four SPTs were identified by these two modalities generating a diagnostic yield of 2.8%. Similarly, 53 p16–negative patients underwent panendoscopy and 35 patients underwent whole-body PET-CT at the time of their diagnosis. Nine SPTs were identified, which corresponded to an overall diagnostic yield of 10.2%. Overall, the diagnostic yield for synchronous SPTs was significantly less in p16-positive patients (p = 0.019) (Table 3).

**Table 3:** Diagnostic yield of various imaging modalities for second primary tumours by p16 status

	**Number of patients**		
	**p16 +**	**p16 -**	**p-value**
Diagnostic work-up			
Whole-body PET-CT	64	35	
Panendoscopy	80	53	
SPTs identified	4	9	
Diagnostic yield	2.8%	10.2%	0.02*

p < 0.05 considered statistically significant.

## Discussion

Several studies have indirectly addressed the issues of second primary tumours in oropharyngeal squamous cell carcinoma. Both Ang et al. and Licitra et al. reported a lower prevalence of SPTs in HPV-positive oropharyngeal squamous cell carcinoma patients, though neither study found statistically significant differences [10,16]. Recent epidemiologic data have also reported a dramatic decline in the incidence of second primary tumours after an index oropharyngeal tumour in the last three decades [17]. Our study comprehensively compares the rate of upper aerodigestive tract second primary tumours in virally mediated OPSCC and non-virally-mediated disease. We report a significantly reduced incidence rate of upper aerodigestive tract second primary tumours in HPV-related OPSCC. This differs from SPTs of non-upper aerodigestive tract sites, which have similar rates between the patient populations. In addition, our study demonstrates a difference in site predilection of SPTs in the head and neck. While p16-positive patients had oral cavity and oropharyngeal involvement, p16-negative patients had a wide distribution of SPTs in the upper aerodigestive tract. Admittedly, the number of second primary tumors are small and firm conclusions regarding site predilection cannot be made.

Interestingly, three HPV-positive patients also had second primary malignancies affecting the lung. The relationship between HPV and lung cancer, particularly in non-smokers is an area of active recent research and remains controversial and poorly understood [18]. However, given the high incidence of co-morbid smoking status in our patients, no causal relationships can be concluded.

**Figure 2:**
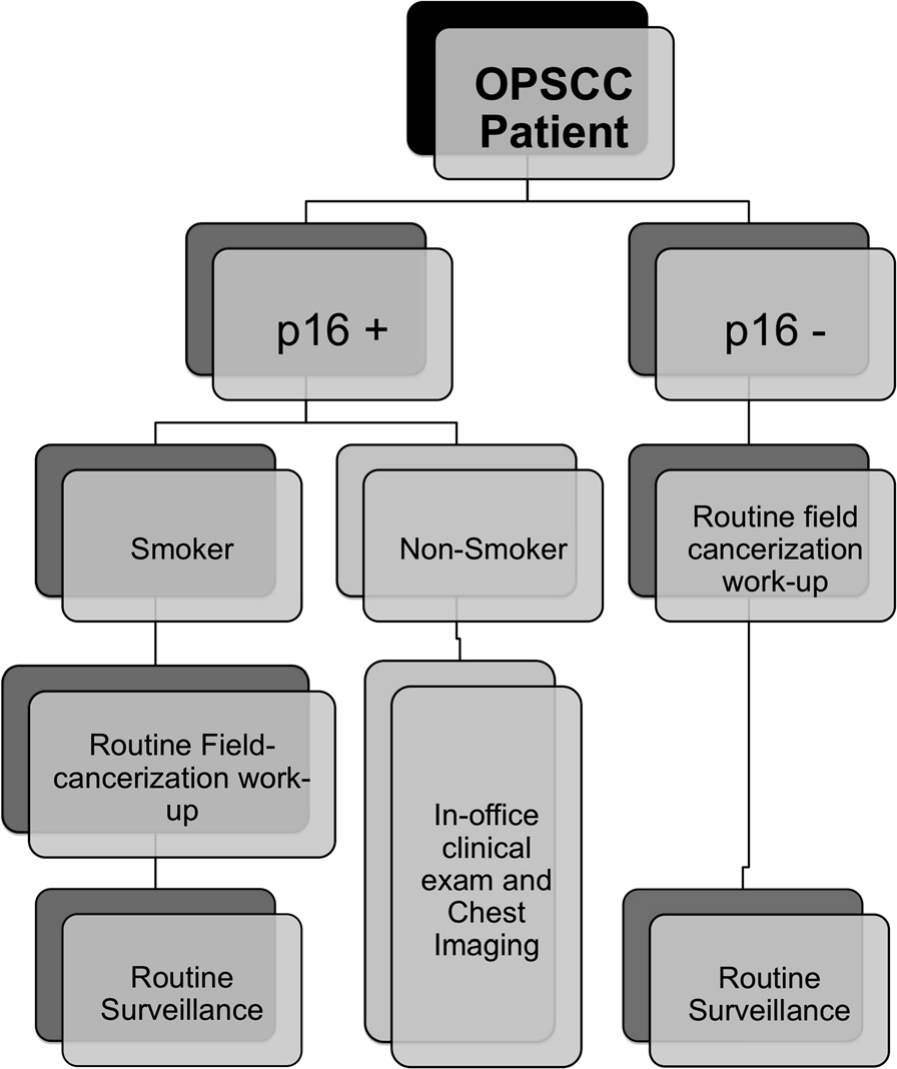
Proposed diagnostic algorithm for second primary tumours by p16 status.

We also examined the diagnostic yield of field cancerization work-up in p16 positive and p16 negative patients. The yield, particularly on panendoscopy was very low in HPV-positive patients. No synchronous second primary tumours were identified on panendoscopy in p16-positive, nonsmokers. In these patients, careful in-office examination, including flexible nasopharyngoscopy, would likely be sufficient for identifying second primaries located in the oral cavity, and oropharynx. In addition, studies have already shown that panendoscopy has limitations in its ability to identify second primary lung malignancies [19]. Whole-body PET-CT on the other hand, has emerged as a cost-effective diagnostic tool [20] for staging and identification of second primary tumours in head and neck cancer [21,22]. Based on our results, a diagnostic pathway for SPTs is proposed in Figure 2. The algorithm acknowledges differences in field cancerization work-up from centre to centre based on practice preferences and resource availability.

This study has a number of limitations. First, retrospective reviews are by nature subject to recall bias, inaccuracies in records, and incomplete data. Smoking status, in particular was reported only as smoker versus life-non-smoker. No differentiation was made between current and former smokers or light and heavy smoking. Secondly, it is possible that patients with virally mediated HNSCC develop second primary tumours much later and thus, this study provides an underestimation of the SPT rates in these patients. Longer longitudinal studies would be needed to confirm that HPV-positive patients remain SPT free beyond a 5 to 10-yr period. Finally, p16 over-expression is not synonymous with the presence of HPV-related cancer but studies that have demonstrated the utility of p16 as a surrogate marker for oncogenic-HPV infection, with up to 100% sensitivity and 93% specificity [23]. P16 status has also been shown to be of more reliable prognostic value and is currently used for clinical stratification of patients in many centers. Thus, our interpretation of p16 status represents the current real-world usage of this marker.

## Conclusions

Patients with HPV-related OPSCC, who are non-smokers have decreased risk of developing second primary tumours in the upper aerodigestive tract and have low yield on field cancerization work-up. This study provides further evidence that virally mediated OPSCC are distinct and may benefit from alternate diagnostic pathways.

## Competing interests

The authors declare that they have no competing interests.

## Authors’ contributions

CCJ, VLB and HS designed the study. VLB and LP performed p16 status analysis. CCJ performed data collection, analysis, and preparation of the manuscript. All authors read and approved the final manuscript.
